# Evaluation of neuropathic-like symptoms and objective signs of neuropathy post-knee replacement in patients with knee osteoarthritis

**DOI:** 10.1016/j.ocarto.2025.100651

**Published:** 2025-07-17

**Authors:** Devin Driscoll, Priyanka Ballal, Na Wang, Laura Frey-Law, Cora E. Lewis, Michael Nevitt, Tuhina Neogi

**Affiliations:** a Section of Rheumatology, Boston University Chobanian & Avedisian School of Medicine, Boston, MA, USA; b Department of Physical Therapy and Rehabilitation, University of Iowa Carver College of Medicine, USA; c Department of Epidemiology, University of Alabama at Birmingham, Birmingham, AL, USA; d University of California San Francisco, San Francisco, CA, USA

**Keywords:** Knee, Osteoarthritis, Replacement, Pain, Neuropathy

## Abstract

**Objective:**

Why persistent pain post-knee replacement occurs is not understood. We examined the association of clinical measures of neuropathy and presence of ‘neuropathic-like pain’ with pain post-knee replacement.

**Design:**

Participants with a knee replacement from the NIH-funded longitudinal cohort Multicenter Osteoarthritis (MOST) Study were examined ∼12-months post-surgery using clinical assessments of neuropathy (von Frey 2 ​g and 26 ​g monofilaments and pin prick) and the painDETECT questionnaire (PDQ; score ≥13 was considered neuropathic-like pain). We evaluated the relation of the clinical neuropathy assessments and the PDQ to achievement of the patient acceptable symptom state (PASS) post-knee replacement using WOMAC, and of the objective clinical neuropathy assessments to neuropathic-like pain on the PDQ using logistic regression.

**Results:**

This cohort study evaluated 171 participants post-knee replacement (mean age 69, 62 ​% female, mean BMI 32.6). Overall, 57 ​% had any abnormality on ≥1 clinical neuropathy assessment (51 ​% hypoesthesia, 10 ​% allodynia/hyperalgesia), and 7 ​% had neuropathic-like pain. There was no association between presence of any abnormality on clinical assessment and not achieving the PASS (OR 1.4, 95 ​% CI 0.5–4.12). In contrast, higher PDQ scores were significantly associated with not achieving the PASS. There was no association between any abnormality on the clinical assessments of neuropathy with PDQ score ≥13 (OR 0.65 (95 ​% CI 0.14–3.02).

**Conclusions:**

Objective clinical assessments of neuropathy were not associated with worse pain status post-knee replacement, while PDQ scores were. PDQ was not associated with clinical assessments of neuropathy. PDQ may be reflective of pain severity in general, and potentially nociplastic, rather than neuropathic, pain.

## 1 Introduction

Knee osteoarthritis (OA) is the most common form of arthritis, characterized by substantial pain and functional limitations. There are limited management option to prevent its progression and consequently, knee replacement is considered definitive treatment for knee OA and the pain associated with it [1]. However, 20–30 ​% of people experience persistent pain post-replacement [2]. The etiology of this persistent pain is unknown, and has been hypothesized that post-surgical neuropathic pain may be a component [2].

If the mechanism of pain in knee OA is purely nociceptive, one would expect resolution following surgery due to removal of the pathologic tissue that had been providing the peripheral nociceptive input. However, because a significant proportion of patients do not have adequate pain relief post-knee replacement this may suggest that persistent pain post-knee replacement may potentially be secondary to some of the tissues that are not resected during surgery providing ongoing peripheral input (e.g., infrapatellar fat pad), as a sequela from the surgical intervention that results in a nerve lesion (neuropathy), or other issue unrelated to the pre-existing OA or surgery. For example, nociplastic pain, which is pain that arises from altered nociception causing central sensitization, in the absence of clear evidence of actual tissue damage or evidence of disease or lesion of the somatosensory system causing the pain [[3], [4], [5]], could be one possible explanation as contributing to persistent pain post-knee replacement.

While neuropathic pain has been hypothesized as an explanation for persistent pain post-knee replacement, the International Association for the Study of Pain definition of neuropathic pain requires a demonstrable lesion or a disease that satisfies neurologic diagnostic criteria [3]. However, to date, studies have not used objective measures of neuropathy to assess the question of etiology of persistent pain post-knee replacement, but rather have primarily relied upon questionnaires that aim to differentiate neuropathic versus nociceptive pain. One such instrument is the painDETECT questionnaire (PDQ), which was developed as a screening tool to differentiate nociceptive pain versus neuropathic pain in patients with chronic low back pain in 2006 [6], specifically mechanical low back pain from radiculopathy. Importantly, the “negative” controls, those deemed to have nociceptive pain only, included those with OA. Based on experts characterizing patients as having neuropathic versus nociceptive pain, the questionnaire was found to have a sensitivity, specificity, and positive predictive value of 85 ​%, 80 ​%, and 83 ​%, respectively [6]. Although patients with OA were classified as having nociceptive pain in the original development, the PDQ was subsequently assessed in a cohort of patients with knee OA in which over 25 ​% were deemed to have neuropathic pain localized to their knees based on a modification of this questionnaire to focus on knee symptoms [7]. Whether they had other conditions that contributed to these findings, such as diabetes or radiculopathy, which are common in OA, is not clear. Therefore, while PDQ has been used as a screening tool for neuropathic pain, studies to date have not reported confirmatory peripheral nerve lesions objectively assessed in relation to PDQ scores in people with OA. An understanding of the mechanism underlying persistent pain post-knee replacement would provide clinical guidance regarding potential therapies to use to ameliorate such symptoms. For example, if there was objective evidence of neuropathy as contributing to persistent pain post-knee replacement, then treatments that target neuropathic pain could be considered for such patients.

The goal of this study was to assess the presence of neuropathy with objective clinical assessments and the PDQ in a cohort of individuals who have undergone knee replacement to examine their relation to persistent pain post-knee replacement.

## 2 Methods

### 2.1 Study sample

We used data from the Multicenter Osteoarthritis (MOST) Study, a NIH-funded longitudinal cohort of older adults who were age 50–79 years at baseline with or at risk of knee OA. Details of the study protocol have been published elsewhere [8]. The study protocol was approved by the institutional review boards at the University of Iowa, University of Alabama, Birmingham, University of California, San Francisco and Boston University School of Medicine. For this cohort study, we used cross-sectional data from an ancillary study of 171 MOST participants who were invited for a follow-up visit 12 months post-knee replacement and were administered the PDQ, objective measures of neuropathy on clinical exam, and pain severity ratings.

### 2.2 Pain detect questionnaire

The PDQ, developed in 2006 in Germany, is a screening questionnaire to determine the presence of neuropathic pain in patients with low back pain [6]. This questionnaire has been extrapolated to other patient populations as a way to screen for the presence of neuropathic pain (or “neuropathic-like” pain). The questionnaire is scored from −1 to 38. A score of −1 to 12 is considered unlikely to have a neuropathic pain component, a score of greater or equal to 13 but less than 19 is considered to be possible neuropathic pain, and a score greater or equal to 19 is considered to be neuropathic pain.

### 2.3 Objective measures of neuropathy

Participants in this ancillary study of the MOST study were assessed 12 months post-knee replacement using standardized somatosensory assessment at the replaced knee per the standard evaluation of pain protocol (StEP protocol) [9]. We used 2 ​g and 26 ​g von Frey monofilaments to evaluate static allodynia or hypoesthesia, and pinprick to evaluate hyperalgesia or hypoesthesia. Each was assessed with four trials. Static allodynia is the sensation of pain for a normally non-painful stimulus, hyperalgesia is an exaggerated pain response to a painful stimulus, and hypoesthesia is no response to non-painful stimulus. Abnormal responses were defined as pain (allodynia or hyperalgesia) or no response (hypoesthesia) if such a response occurred in ≥3/4 trials.

### 2.4 Outcome: patient acceptable symptom state

Pain severity was assessed using the Western Ontario and McMaster Universities Osteoarthritis Index (WOMAC) pain subscale (range 0–20; higher scores indicate more pain severity) [10]. We defined the presence of pain persistence post-knee replacement based upon the patient acceptable symptom state (PASS) for WOMAC. PASS is a clinically relevant cutoff score which defines an “acceptable state” for the patient [[11], [12], [13], [14]]. A score equal to or less than PASS can be defined as the patient feeling well, while a score greater than PASS exceeds the highest level of symptoms beyond which the patient considers themselves to be well. Based upon published thresholds for the WOMAC pain subscale, we defined PASS to be less than or equal to 5/20 for patients with knee OA [11,13,14].

### 2.5 Analytic approach

We assessed the descriptive statistics with frequency and mean with standard deviations as appropriate for participant characteristics, prevalence of pain severity, objective tests of neuropathy on bedside exam, and PDQ score. We defined neuropathic-like pain as a PDQ score ≥13 (we were unable to analyze a PDQ score ≥19 due to lack of sufficient participants with a score above this threshold). We assessed the relation of the clinical neuropathy assessments, PDQ score (per SD unit increase), and of neuropathic-like pain on the PDQ to pain persistence post-knee replacement (defined as not achieving the PASS). We also assessed the relation of the objective clinical neuropathy assessments to neuropathic-like pain on the PDQ using logistic regression. We adjusted for potential confounders including age, sex, BMI, diabetes, and depressive symptoms in each model. In sensitivity analyses, we also adjusted for pain medications (e.g., NSAIDs, opioids, centrally acting agents such as duloxetine).

## 3 Results

Our study sample of 171 participants had a mean age of 69 years and the majority were obese, white, and female, with a mean BMI of 32.6 (Table 1). Additionally, 17 ​% of the study sample had diabetes.

**Table 1:** Mean and frequency of patient characteristics, PDQ, pain severity rating, and clinical objective assessments of neuropathy.

Clinical characteristics	Descriptive (n ​= ​171)
Age, year (mean ​± ​SD)	69.0 ​± ​7.8
Female (%)	62 ​%
Race (%) White	87 ​%
Other	13 ​%
BMI (mean ​± ​SD)	32.6 ​± ​6.9
Diabetes (%)	17 ​%
PDQ (mean ​± ​SD)	4.4 ​± ​4.7
PDQ≥13 (%)	7.0 ​%
WOMAC (mean ​± ​SD)	2.4 ​± ​3.3
Pain persistence post-knee replacement (defined as not achieving the WOMAC PASS) (%)	15.2 ​%
Hypoesthesia on any modality (%)	51.2 ​%
2 ​g von Frey monofilament	47.3 ​%
26 ​g von Frey monofilament	14.7 ​%
Pin prick	18.6 ​%
Allodynia or hyperalgesia on any modality (%)	10.1 ​%
2 ​g von Frey monofilament	0.0 ​%
26 ​g von Frey monofilament	4.7 ​%
Pin prick	9.3 ​%
Any abnormality (allodynia, hyperalgesia, or hypoesthesia) on any modality (%)	56.6 ​%

The mean PDQ score was 4.4 (SD 4.7) and 7 ​% of the participants had a PDQ greater or equal to 13 (i.e., neuropathic-like pain) 12 months post-knee replacement. The mean WOMAC score was 2.4 (SD 3.3) and 15.2 ​% did not meet PASS for WOMAC (i.e., had persistent pain post-knee replacement) (Table 1).

For objective measures of neuropathy using clinical assessments, over half (56.6 ​%) had an abnormal response (pain or no response) in ≥3/4 trials to any of the clinical assessments (2 ​g von Frey, 26 ​g von Frey, or pinprick). Allodynia or hyperalgesia in response to any of the modalities was present in 10.1 ​%, while hypoesthesia was present in 51.2 ​%. The frequency of participants with abnormal responses in terms of either pain (allodynia or hyperalgesia) or no response (hypoesthesia) in ≥3/4 trials to each of the individual clinical assessments were as follows: for 2 ​g von Frey monofilament, 0 ​% had allodynia and 47.3 ​% had hypoesthesia; for 26 ​g von Frey monofilament, 4.7 ​% had allodynia and 14.7 ​% had hypoesthesia; and for pinprick, 9.3 ​% had hyperalgesia and 18.6 ​% had hypoesthesia (Table 1).

The presence of any abnormality on any of the clinical assessments was not statistically significantly associated with pain persistence post-knee replacement (defined as not achieving PASS), with an OR of 1.43 (95 ​% CI 0.50–4.12; Table 2). Further adjustment for pain medications did not meaningfully alter the effect estimates (Supplemental Table 1). None of the individual clinical assessments of neuropathy were associated with pain persistence post-knee replacement except for hypoesthesia with pin prick (OR 3.85, 95 ​% CI 1.15–12.9; Supplemental Table 3). In contrast, both higher PDQ score and neuropathic-like pain on the PDQ (i.e., score greater or equal to 13) were significantly associated with likelihood of not achieving PASS with an OR of 2.09 (95 ​% CI 1.38–3.14) per SD unit increase in PDQ and 6.13 (95 ​% CI 1.61–23.4) for PDQ score greater or equal to 13 (Table 2). These point estimates were amplified when pain medications were included in the adjustment (Supplemental Table 1).

**Table 2:** Relation of clinical assessments of neuropathy and of painDETECT to pain persistence post-knee replacement (defined as not achieving the Patient Acceptable Symptom State post-knee replacement).

Assessment of ‘neuropathy’:	aOR (95 ​% CI) for pain persistence post-knee replacement^b^
painDETECT score per SD unit increase	2.09 (1.38–3.14), p ​= ​0.0004
painDETECT score ≥13 (“neuropathic-like pain”)	6.13 (1.61–23.4), p ​= ​0.008
Any abnormality (allodynia/hyperalgesia or hypoesthesia) on clinical assessments of neuropathy:^a^	
Any modality	1.43 (0.50–4.12), p ​= ​0.5
2 ​g von Frey monofilament	1.72 (0.62, 4.80), p ​= ​0.3
26 ​g von Frey monofilament	0.83 (0.23, 2.96), p ​= ​0.8
Pin prick	2.10 (0.74, 5.90), p ​= ​0.2

a Defined as either: pain response (allodynia/hyperalgesia) in ≥3/4 trials OR no response (hypoesthesia) in ≥3/4 trials to the clinical assessments (von Frey monofilaments (2 ​g, 26 ​g) or pinprick).

b Adjusted for age, sex, BMI, diabetes, and depressive symptoms.

There was no relation of PDQ to any abnormality on clinical assessments of neuropathy, whether PDQ was assessed as a continuous score or dichotomized as “neuropathic-like” pain (score ≥13), with ORs of 1.13 (95 ​% CI 0.75–1.72) and 0.65 (95 ​% CI 0.14–3.02), respectively (Table 3). Similar results were found when pain medications were included in the adjustment (Supplemental Table 2). Additionally, PDQ score was not associated with individual modalities (Supplemental Table 4), including hypoesthesia with pin prick or 26 ​g von Frey monofilament because none of the participants who had hypoesthesia with pin prick or 26 ​g von Frey monofilament had a PDQ score in the neuropathic-like pain range.

**Table 3:** Relation of painDETECT to any abnormality on clinical assessments of neuropathy.

painDETECT Score	aOR (95 ​% CI) for association with any abnormality on clinical assessment of neuropathy^a^^,^^b^
painDETECT score per SD unit increase	1.13 (0.75–1.72), p ​= ​0.5
painDETECT score ≥13 (“neuropathic-like pain”)	0.65 (0.14–3.02), p ​= ​0.6

a Defined as either: pain response in ≥3/4 trials OR no response in ≥3/4 trials to any one of the clinical assessments (von Frey monofilaments (2 ​g, 26 ​g) or pinprick).

b Adjusted for age, sex, BMI, diabetes, and depressive symptoms.

## 4 Discussion

We found that objective clinical bedside exam findings of potential neuropathy (allodynia, hyperalgesia, or hypoesthesia) were not associated with persistent pain post-knee replacement with the exception of hypoesthesia with pin prick. In contrast, higher PDQ scores or neuropathic-like pain on the PDQ were associated with having persistent pain post-knee replacement. Additionally, PDQ scores, including in the ‘neuropathic-like’ range, were not associated with objective bedside clinical exam findings of neuropathy, including the one clinical exam finding of pin prick hypoesthesia which was associated with pain persistence, raising questions about what PDQ scores are reflecting in people with knee OA.

Persistent pain post-knee replacement has a large public health burden given that knee replacement is a common surgical procedure, with OA being the most common indication for this procedure [15]. Potential causes for pain have been cited to include neuropathy, arthrofibrosis, aseptic loosening, avascular necrosis, central sensitization, component malpositioning, infection, instability, overstuffing, patellar maltracking, polyethylene wear, psychological factors and unresurfaced patella [15]. Our finding of an association between pin prick hypoesthesia and pain persistence post-knee replacement suggests potential contribution of neuropathy to this phenomenon. It is unclear as to whether this finding may be surgery-related since we do not have an evaluation of these measures prior to their knee replacement. Of note, only one-third of those with pin prick hypoalgesia had diabetes.

We are unaware of other studies that have formally evaluated clinical measures of neuropathy and the painDETECT in a post-knee replacement sample. The relationship between knee OA (i.e., not knee replacement) and neuropathic pain defined by painDETECT was initially examined by Hochman et al. [7]. They found 28 ​% of their cohort had neuropathic symptoms on painDETECT [7]. Because participants with bilateral knee replacement were excluded from the study and the index knee was a non-replaced knee, their study results are not directly comparable to the current study. While Hochman et al. used another neuropathic pain questionnaire to evaluate the construct validity of the painDETECT, they did not compare the results of painDETECT to objective measures of neuropathy on exam, and some of their findings from painDETECT may potentially reflect comorbid diabetes or radiculopathy which are common in people with OA.

In our study, over half of the participants (56.6 ​%) had some form of abnormal response to the beside clinical exam testing, including allodynia or hyperalgesia responses as well as hypoesthesia responses to the three modalities tested (2 ​g and 26 ​g von Frey monofilament, and pin prick). With the exception of pin prick hypoesthesia, the clinical exam findings were not associated with worse pain status post-knee replacement, defined as not achieving the PASS for WOMAC. Thus, while many participants may have had clinical evidence of neuropathy, the majority of these findings did not appear to explain pain-persistence post-KR.

In contrast, the PDQ was associated with pain persistence post-KR. The PDQ, which was originally developed as a screening questionnaire to determine the prevalence of neuropathic pain component in patients with low back pain has been extrapolated to patients with OA, including knee OA [6]. However, in this current study, the PDQ, particularly in the ‘neuropathic-like pain’ range, was not associated with any of the findings on the clinical assessments of neuropathy, including the one clinical exam finding of neuropathy that did appear to be associated with pain persistence post-knee replacement.

Questionnaires are more feasible to use in clinical studies and clinical care than performing objective assessments for findings of neuropathy. However, use of questionnaires alone to identify neuropathic pain in a condition like knee OA may not be ideal given that symptoms may be reflective of altered nervous system functioning, such as central sensitization and nociplastic pain, rather than a true neuropathy [16]. This can lead to misclassification of a patient's pain as neuropathic if based on clinical symptoms alone [16], which would have clinical implications if one were to consider using treatments directed towards neuropathic pain to manage pain persistence post-knee replacement. Multiple studies have also shown that PDQ may be a measure of central sensitization, rather than neuropathic pain, with high scores on PDQ having been associated with greater odds of central sensitization at both the knee and the hip [[17], [18], [19], [20]]. Prior work using the MOST cohort also showed that objective measures of central sensitization were associated with pain severity post-knee replacement [21]. Therefore, higher PDQ scores may represent more than a single mechanism of pain and may more generally reflect the degree of pain severity. For example, in a study by Kim et al., central sensitization, as measured by the Central Sensitization Inventory, and neuropathic-like pain, as measured by PDQ, found that participants with both central sensitization and ‘neuropathic-like pain’ had worse pain severity compared with those who had either condition alone or without either condition [20].

Limitations of our study should be acknowledged. We did not perform EMG/NCS to evaluate presence of neuropathy more definitively. Nonetheless, the bedside tools utilized are used clinically to assess for neuropathy and have been demonstrated to be useful in differentiating neuropathic pain from other causes [9]. The mean pain severity was not high in this sample; however, ∼15 ​% of our sample did not achieve a PASS, indicating clinically relevant pain persistence post-KR, and is of a similar prevalence to that reported in the literature. The study sample was predominantly white, and therefore our findings may not be generalizable to other groups. Our study also had strengths. We enrolled participants into our study irrespective of symptoms rather than focusing solely on those with post-KR pain persistence. We also accounted for presence of diabetes, a common cause of neuropathy and a common comorbidity in knee OA, which hadn't been consistently done in prior studies of knee OA utilizing the PDQ. Importantly, this study provides the first direct comparison of a set of bedside tools for assessment of neuropathy with the PDQ.

## 5 Conclusion

In summary, we found that clinical assessments of neuropathy were generally not associated with persistent pain post-knee replacement, with the exception of pin prick hypoesthesia, nor with the PDQ, including scores indicating ‘neuropathic-like’ pain. Thus, the mechanism underlying post-knee replacement pain persistence in this sample does not appear to be predominantly related to clinically evident neuropathy. In contrast, higher painDETECT scores, including in the ‘neuropathic-like’ pain range, were associated with pain persistence post-KR but not with any of the clinical assessments of neuropathy. Thus, the PDQ does not appear to be specific for neuropathy in this clinical setting. It is therefore possible that the PDQ is reflective of pain severity in general, and potentially nociplastic pain, rather than neuropathic pain, in post-knee replacement pain persistence.

## Author’s contributions

Devin Driscoll MD- Concept and design, Interpretation of data, Drafting of the article, Final Approval, Responsibility for the integrity of the work as a whole.

Priyanka Ballal- Concept and design, Interpretation of data, Critical revision, Final approval.

Na Wang PhD- Analysis, Statistical expertise, Revising it critically for important intellectual content, Final approval.

Laura Frey-Law- Interpretation of data, Critical revision, Provision of study materials, Collection and assembly of data, Final approval.

Beth Lewis- Obtaining of funding, Interpretation of data, Critical revision, Provision of study materials/patients, Collection and assembly of data, Final approval.

Michael Nevitt PhD- Obtaining of funding, Interpretation of data, Critical revision, Provision of study materials/patients, Collection and assembly of data, Final approval.

Tuhina Neogi MD PhD- Obtaining of funding, Concept and design, Interpretation of data, Drafting of the article, Critical revision of the article for important intellectual content, Provision of study materials/patients, Collection and assembly of data, Final Approval, Responsibility for the integrity of the work as a whole.

## Funding

NIH R01 AR062506, NIH P30 AR072571, NIH K24 AR070892, NIH U01 AG18820, NIH U01 AG18832, NIH U01 AG18947, NIH U01 AG19069, NIH U19 AG076471.

## Declaration of competing interest

Devin Driscoll MD-none.

Priyanka Ballal MD-none.

Na Wang PhD-none.

Laura Frey-Law-none.

Beth Lewis MD-none.

Michael Nevitt PhD-none.

Tuhina Neogi MD PhD-none.
