# Supplementary material for: Evaluation of neuropathic-like symptoms and objective signs of neuropathy post-knee replacement in patients with knee osteoarthritis
Source: Osteoarthr Cartil Open. 2025 Jul 17;7(3):100651. doi: 10.1016/j.ocarto.2025.100651 (PMC12305714; doi:10.1016/j.ocarto.2025.100651)
Supplement: Multimedia component 1 [file mmc1.docx]

**Supplemental Materials**

**Supplemental Table 1: Relation of clinical assessments of neuropathy and of painDETECT to pain persistence post-knee replacement (defined as not achieving the Patient Acceptable Symptom State post-knee replacement) with adjustment including pain medications**

| Assessment of ‘neuropathy’: | aOR (95% CI) for pain persistence post-knee replacement^Ω^ |
| --- | --- |
| painDETECT score per SD unit increase | 2.44 (1.56-3.84), p=0.0001 |
| painDETECT score ≥13 (“neuropathic-like pain”) | 10.3 (2.41-44.4), p=0.002 |
| Any abnormality (allodynia/hyperalgesia or hypoesthesia) on clinical assessments of neuropathy:* |  |
| Any modality | 1.16 (0.39-3.49), p=0.8 |
| 2g von Frey monofilament | 1.44 (0.49-4.20), p=0.5 |
| 26g von Frey monofilament | 0.90 (0.25-3.28), p=0.9 |
| Pin prick | 2.03 (0.70-5.89), p=0.2 |

*Defined as either: pain response (allodynia/hyperalgesia) in ≥3/4 trials OR no response (hypoesthesia) in ≥3/4 trials to the clinical assessments (von Frey monofilaments (2g, 26g) or pinprick)

^Ω^Adjusted for age, sex, BMI, diabetes, depressive symptoms, and pain medications

**Supplemental Table 2: Relation of painDETECT to any abnormality on clinical assessments of neuropathy with adjustment including pain medications**

| painDETECT Score | aOR (95% CI) for association with any abnormality on clinical assessment of neuropathy*^Ω^ |
| --- | --- |
| painDETECT score per SD unit increase | 1.39 (0.72-2.70), p=0.3 |
| painDETECT score ≥13 (“neuropathic-like pain”) | 0.95 (0.16-5.61), p=0.96 |

*Defined as either: pain response in ≥3/4 trials OR no response in ≥3/4 trials to any one of the clinical assessments (von Frey monofilaments (2g, 26g) or pinprick)

^Ω^Adjusted for age, sex, BMI, diabetes, depressive symptoms, and pain medications

**Supplemental Table 3: Relation of clinical assessments of neuropathy to likelihood of not achieving the Patient Acceptable Symptom State post-knee replacement**

| Assessment | aOR (95% CI) for not achieving the WOMAC PASS post-knee replacement | aOR (95% CI) for not achieving the WOMAC PASS post-knee replacement adjustment with pain medication |
| --- | --- | --- |
| Hypoesthesia on any modality^#^ (%) | 1.47 (0.53, 4.10), p=0.5 | 1.22 (0.42, 3.58), p=0.7 |
| 2g von Frey monofilament | 1.72 (0.62, 4.80), p=0.3 | 1.44 (0.49, 4.20), p=0.5 |
| 26g von Frey monofilament | 0.89 (0.21, 3.72), p=0.9 | 0.97 (0.23, 4.08), p=0.97 |
| Pin prick | 3.85 (1.15, 12.9), 0.03 | 3.34 (0.97, 11.5), p=0.06 |
| Allodynia or Hyperalgesia on any modality^^^ (%) | 0.67 (0.11, 4.04), p=0.7 | 0.75 (0.12, 4.62), p=0.8 |
| 2g von Frey monofilament | N/A* | N/A* |
| 26g von Frey monofilament | 0.54 (0.04, 7.09), p=0.6 | 0.60 (0.04, 8.60), p=0.7 |
| Pin prick | 0.72 (0.12, 4.38), p=0.7 | 0.83 (0.13, 5.24), p=0.8 |

^#^Defined as no response in ≥3/4 trials to stimuli

^^^Defined as pain response in ≥3/4 trials to stimuli

*Not applicable because there were no participants with an allodynia response to 2g von Frey monofilament

**Supplemental Table 4: Relation of clinical assessments of neuropathy to painDETECT**

| Assessment: | aOR (95% CI) for painDETECT score per SD unit increase | aOR (95% CI) for painDETECT score ≥13 (“neuropathic-like pain”) |
| --- | --- | --- |
| Hypoesthesia on any modality^#^ (%) | 1.08 (0.72, 1.63), p=0.7 | 0.80 (0.17, 3.70), p=0.8 |
| 2g von Frey monofilament | 1.11 (0.74, 1.66), p=0.6 | 0.85 (0.18, 3.99), p=0.8 |
| 26g von Frey monofilament | 1.16 (0.63, 2.13), p=0.6 | N/A* |
| Pin prick | 1.05 (0.59, 1.85), p=0.9 | N/A* |
| Allodynia or Hyperalgesia on any modality^^^ (%) | 1.11 (0.58, 2.11), p=0.8 | 1.26 (0.12, 13.1), p=0.8 |
| 2g von Frey monofilament | N/A** | N/A** |
| 26g von Frey monofilament | 1.84 (0.80, 4.20), p=0.2 | 5.59 (0.35, 89.7), p=0.2 |
| Pin prick | 1.07 (0.54, 2.10), p=0.8 | 1.36 (0.13, 14.3), p=0.8 |

^#^Defined as no response in ≥3/4 trials to stimuli

^^^Defined as pain response in ≥3/4 trials to stimuli

*Not applicable because no participant with hypoesthesia to pin prick or 26g von Frey monofilament had a PDQ score ≥13

**Not applicable because there were no participants with an allodynia response to 2g von Frey monofilament
